# The Rural Opioid Initiative Consortium description: providing evidence to Understand the Fourth Wave of the Opioid Crisis

**DOI:** 10.1186/s13722-022-00322-5

**Published:** 2022-07-26

**Authors:** Richard A. Jenkins, Bridget M. Whitney, Robin M. Nance, Todd M. Allen, Hannah L. F. Cooper, Judith Feinberg, Rob Fredericksen, Peter D. Friedmann, Vivian F. Go, Wiley D. Jenkins, P. Todd Korthuis, William C. Miller, Mai T. Pho, Abby E. Rudolph, David W. Seal, Gordon S. Smith, Thomas J. Stopka, Ryan P. Westergaard, April M. Young, William A. Zule, Joseph A. C. Delaney, Judith I. Tsui, Heidi M. Crane, the Rural Opioid Initiative

**Affiliations:** 1 Prevention Research Branch, National Institute on Drug Abuse, 3WFN MSC 6024, 301 North Stonestreet Ave, Bethesda, MD 20892 USA; 2 University of Washington Harborview Medical Center, 325 9th Ave, Box 359931, Seattle, WA 98106 USA; 3 Ragon Institute of MGH, MIT and Harvard, Rm 764 400 Technology Square, Cambridge, MA 02139 USA; 4 Rollins School of Public Health, Emory University, Grace Crum Rollins Building 1518 Clifton Road, Atlanta, GA 30322 USA; 5 West Virginia University, 930 Chestnut Ridge Road, PO Box 9156, Morgantown, WV 26505 USA; 6 Baystate Medical Center—University of Massachusetts, Office of Research, UMass Chan Medical School - Baystate, 3601 Main Street, 3rd Floor, Springfield, MA 01199 USA; 7 University of North Carolina—Chapel Hill, 363 Rosenau Hall CB# 7440, Chapel Hill, NC 27599 USA; 8 Southern Illinois University, 201 E Madison Street, Springfield, IL 62702 USA; 9 Oregon Health & Science University, 3270 Southwest Pavilion Loop OHSU Physicians Pavilion, Suite 350, Portland, OR 97239 USA; 10 The Ohio State University, 302 Cunz Hall 1841 Neil Ave, Columbus, OH 43210 USA; 11 University of Chicago, 5841 S. Maryland Avenue, Chicago, IL 60637 USA; 12 Department of Epidemiology and Biostatistics, Temple University College of Public Health, 1301 Cecil B Moore Avenue, Ritter Annex 905, Philadelphia, PA USA; 13 Tulane University, 1440 Canal Street, Suite 2210, New Orleans, LA 70112 USA; 14 Tufts University School of Medicine Public Health and Community Medicine, 136 Harrison Avenue, Boston, MA 02111 USA; 15 University of Wisconsin-Madison, 1685 Highland Avenue, 5th Floor, Madison, WI 53705-2281 USA; 16 University of Kentucky, 760 Press Avenue Suite 280, Lexington, KY 40536 USA; 17 RTI International, 3040 E. Cornwallis Road, PO Box 12194, Research Triangle Park, NC 2709-2194 USA; 18 College of Pharmacy, University of Manitoba, Apotex Centre, 750 McDermot Ave. W, Winnipeg, MB R3E 0T5 Canada

**Keywords:** Opioids, Methamphetamine, Rural, Substance use, Injection drug use, Overdose

## Abstract

**Objective:**

To characterize and address the opioid crisis disproportionately impacting rural U.S. regions.

**Methods:**

The Rural Opioid Initiative (ROI) is a two-phase project to collect and harmonize quantitative and qualitative data and develop tailored interventions to address rural opioid use. The baseline quantitative survey data from people who use drugs (PWUD) characterizes the current opioid epidemic (2018–2020) in eight geographically diverse regions.

**Results:**

Among 3,084 PWUD, 92% reported ever injecting drugs, 86% reported using opioids (most often heroin) and 74% reported using methamphetamine to get high in the past 30 days; 53% experienced homelessness in the prior 6 months; and 49% had ever overdosed. Syringe service program use varied by region and 53% had ever received an overdose kit or naloxone prescription. Less than half (48%) ever received medication for opioid use disorder (MOUD).

**Conclusions:**

The ROI combines data across eight rural regions to better understand drug use including drivers and potential interventions in rural areas with limited resources. Baseline ROI data demonstrate extensive overlap between opioid and methamphetamine use, high homelessness rates, inadequate access to MOUD, and other unmet needs among PWUD in the rural U.S. By combining data across studies, the ROI provides much greater statistical power to address research questions and better understand the syndemic of infectious diseases and drug use in rural settings including unmet treatment needs.

## Introduction

The 2014–2015 HIV outbreak among people who inject drugs (PWID) in Scott County, Indiana [1], as well as the increased hepatitis C virus (HCV) incidence in rural areas across the U.S [2], magnified the need to understand the risk environment and develop public health interventions that prevent infectious diseases and other harms related to drug use in rural settings where access to evidence-based treatment and prevention may be more limited. There has.

been little systematic multi-site research on injection drug use (IDU) or opioid use disorders in rural areas [3], although some literature characterizes rural methamphetamine [4], prescription opioid, and heroin use in relatively small population centers [5–7], scarcity of sterile syringes [8], and risks of overdose and patterns of substance use in Appalachia [9]. Subsequent modeling suggested that many rural counties are vulnerable to HIV and/or HCV epidemics related to opioid injection [10].

The Rural Opioid Initiative (ROI) developed as a response to this need to better understand and address the rural opioid crisis in the United States (U.S.) through an internal 2015 National Institute on Drug Abuse (NIDA) proposal and a series of meetings with representatives from the Centers for Disease Control and Prevention (CDC), Substance Abuse and Mental Health Administration (SAMHSA), and Appalachian Regional Commission (ARC) in 2016 [11]. Agency representatives recognized that data availability, resources and policies varied by state and within states by locality, and that these would influence implementation, adoption and effectiveness of evidence-based harm reduction interventions that had been tested in urban settings. Agency representatives also recognized that adoption and effective implementation of interventions were more likely if local leaders recognized characteristics of their own communities’ social and policy environments [12]. Consequently, the ROI adopted a two-phase design whereby projects would collect local data and conduct research to fill data gaps over a two-year period. Those data were used to inform development of demonstration-type projects in phase two that would be implemented over three years.

This paper summarizes the design, methodology, and baseline description of the ROI cohort of people who use drugs (PWUD) to characterize and better inform treatment and other interventions for the current opioid crisis in geographically diverse rural regions of the U.S.

## Methods

### Overview

The ROI recruited PWUD from rural regions—a historically understudied population. The ROI includes 8 studies spanning 10 states and 65 U.S. counties. Studies are conducting research in two phases; the first phase involved epidemiologic and policy scans and the collection and harmonization of qualitative and quantitative data to permit comparisons and data aggregation across sites. The second phase focuses on interventions. This paper describes data collection methods and presents cross-sectional quantitative data from phase one.

### Study eligibility

**Fig. 1:**
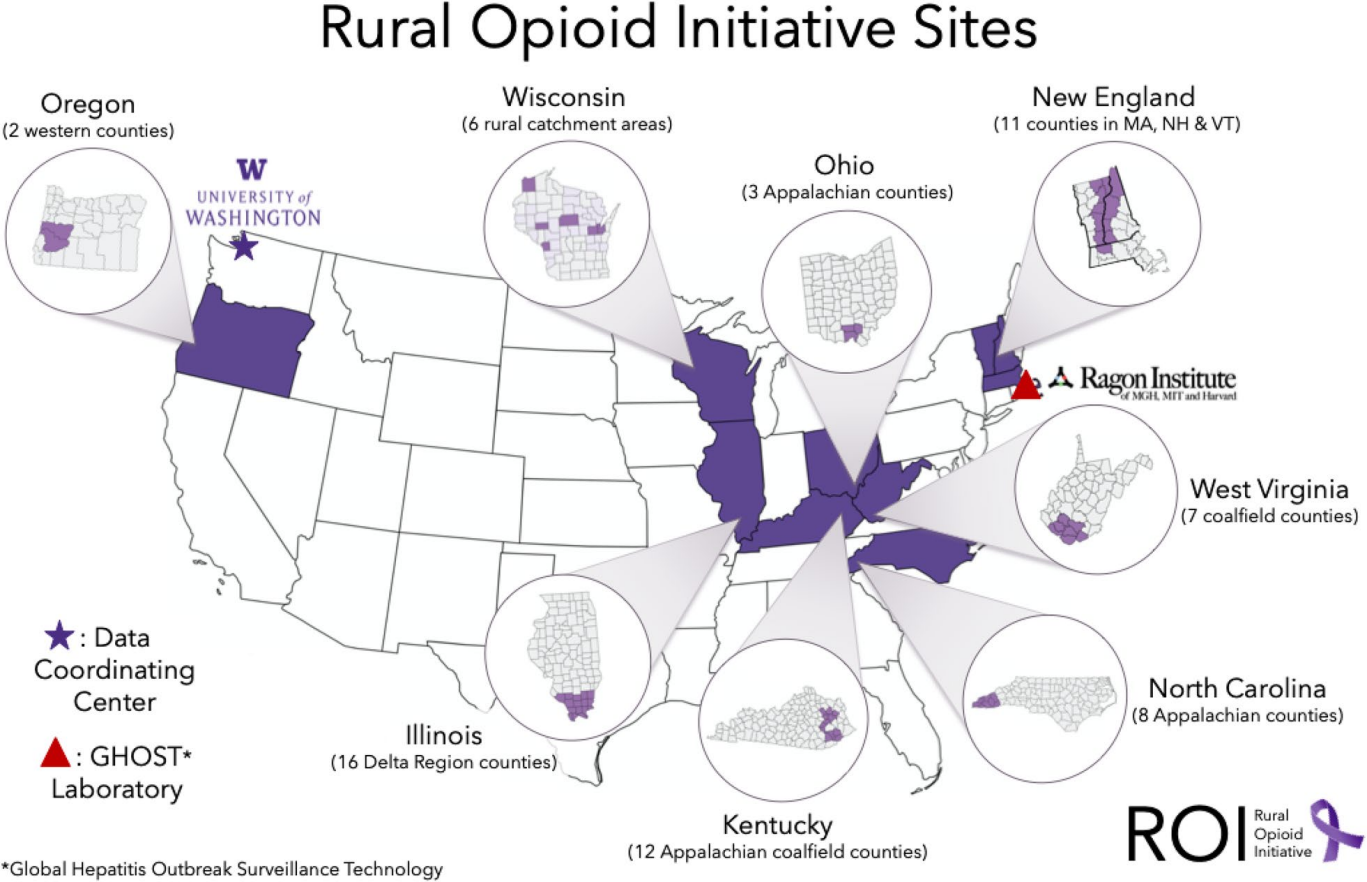
Location of studies in the Rural Opioid Initiative

Studies were required to demonstrate rurality in addition to an ability to collect data on opioid overdoses, infectious diseases and other relevant domains. Government definitions of rural vary by federal agency, which can affect population estimates and program offerings [13]. These differences are summarized on the Health Resources and Services Administration (HRSA) “Am I Rural” website [14]. Funding agencies used this website to confirm rurality and other indicators of vulnerability. Funders permitted small metropolitan areas, micropolitan areas, and metropolitan areas of < 250,000 persons to apply for funding under this initiative, as some rural communities with significant opioid-related and intertwined HIV, HCV and overdose epidemics (e.g., Scott County, Indiana [1]) are classified in metropolitan statistical areas. Thus, ROI study sites include small cities like Portsmouth, Ohio and Keene, New Hampshire; county seats with small colleges that serve as trading hubs like Morehead, Kentucky; former coal mining hubs in West Virginia and Kentucky and a variety of small towns in New England, central Appalachia, southern Illinois, western North Carolina, Wisconsin, and Oregon. Figure 1 shows the diverse geography of the 8 participating studies (Illinois: IL; Kentucky: KY; North Carolina: NC; New England: NE; Ohio: OH; Oregon: OR; Wisconsin: WI; and West Virginia: WV).

### Project design

ROI studies were structured as two-phase awards with phase one focused on building local collaborations, aggregating local data, collecting additional data to fill data gaps, and harmonizing core data elements across study sites. The second phase (currently in progress) focuses on sustainable, locally tailored intervention projects informed by phase one collaborations and assessments. All sites obtained local IRB approval for activities and data-sharing within the ROI.

### ROI management structure

The ROI is a collaboration between: (1) individual study teams responsible for conducting and monitoring enrollment and data collection at their sites; (2) scientific officers and other representatives from federal funding agencies (NIDA, CDC, SAMHSA, ARC) who offer guidance and the federal perspective on the project and its aims; (3) the Data Coordinating Center (DCC) which includes researchers who facilitate cross-site data linkages, provides methodologic and analytic support for quantitative and qualitative data, and facilitates working groups and annual meetings; and (4) the chair of the steering committee, Dr. Holly Hagan, an independent academic researcher who provides expertise and advice. Working Groups focus on specific aspects of study methods and implementation, including quantitative and qualitative data collection, analysis, and field operations. The steering committee includes the chair, site principal investigators, scientific officers and the DCC team.

### Participant recruitment and eligibility

Participants were recruited between January 2018 and March 2020 (exact dates varied by study). Eligible individuals had to report past 30-day use of any opioid “to get high” (heroin, prescription pain medication) and/or past 30-day injection of any drug. Eligibility criteria were tailored slightly to meet region-specific needs (Table 1). Three studies required IDU verified by the presence of lesions at injection sites or the ability to demonstrate knowledge of drug preparation and injection techniques. Two studies that did not require IDU collected urine specimens that were tested for the presence of opioid metabolites. The minimum age was 15 in two studies and 18 in the others. All studies had local human subjects approval.

**Table 1:** Description of studies in the Rural Opioid Initiative, 2018–2020

Study	N	Region	Target Population	Individual inclusion and exclusion criteria
Ending Transmission of HIV, HCV, and STDs and Overdose in Rural Communities of People Who Inject Drugs (ETHIC)	173	16 Delta Regional Authority Counties in Southern Illinois	PWID and/or people who use opioids to get high	Injection of any drug to get high or non-injection use of opioids to get high in the past 30 days; ≥ 15 years of age; resident of study area
Kentucky Communities and Researchers Engaging to Halt the Opioid Epidemic (CARE2HOPE)	338	12 Appalachian Counties in Eastern Kentucky (with survey data collection from people who used drugs focused on 5 of the 12 counties)	PWID and/or people who use opioids to get high	Injection of any drug to get high or non-injection use of opioids to get high in the past 30 days; ≥ 18 years of age; resident of study area
Mitigating the Outcomes Associated with the Injection Drug Use Epidemic in Southern Appalachia (SA-TLC)	350	8 Appalachian Counties in Western North Carolina	PWID and/or people who use opioids to get high	Injection of any drug to get high or non-injection use of opioids to get high in the past 30 days; ≥ 18 years of age; resident of study area and intent to stay in study area for at least 12 months
Drug Injection Surveillance and Care Enhancement for Rural Northern New England (DISCERNNE)	589	11 Counties along the I-91/CT River Corridor in Massachusetts, New Hampshire, and Vermont	PWID and/or people who use opioids to get high	Injection of any drug to get high or non-injection use of opioids to get high in the past 30 days; ≥ 18 years of age; resident of study area
Implementing a Community-Based Response to the Opioid Epidemic in Rural Ohio	258	3 Appalachian Counties in Southeastern Ohio	PWID and/or people who use opioids to get high	Injection of any drug to get high or non-injection use of opioids to get high in the past 30 days; ≥ 18 years of age; resident of study area
Oregon HIV/Hepatitis and Opioid Prevention and Engagement (OR-HOPE) Study	174	2 Southwestern Counties in Oregon	PWID and/or people who use opioids to get high	Injection of any drug to get high or non-injection use of opioids to get high in the past 30 days; ≥ 18 years of age; resident of study area
Community-Based, Client-Centered Prevention Homes to Address the Rural Opioid Epidemic	991	Northern Wisconsin counties (6 rural catchment areas)	PWID	Injection of any drug to get high in the past 30 days; ≥ 15 years of age; resident of study area
Rural West Virginia Responds to Opioid Injection Epidemics: From Data to Action	175	7 Coalfield Counties in Southern West Virginia	PWID and/or people who use opioids to get high	Injection of any drug to get high or non-injection use of opioids to get high in the past 30 days; ≥ 18 years age; resident of study area

*CT* Connecticut, *PWID* people who inject drugs

Respondent-driven sampling (RDS) was used [15] to ensure inclusion of potentially hard-to-reach participants from rural regions. Each study site enrolled between 42 and 279 “seeds” to initiate peer recruitment. Seeds were recruited from syringe service programs (SSPs), local health departments, community health centers, other relevant agencies and street outreach. Seeds were given up to six coupons to recruit peers (maximum number varied by study). Each enrolled non-seed peer recruit could recruit up to five eligible peers (maximum number varied by study). Financial incentives were offered for recruitment ($10-$20 per eligible peer) and for study participation ($40-$60).

### Quantitative data collection

All studies administered a standardized survey at the enrollment interview; five studies administered it using a centrally-developed Audio Computer-Assisted Self-Interview (ACASI), two used Computer-Assisted Self-Interviews (CASI) in REDCap, and one involved interviewer-administration of Computer-Assisted Personal Interviews (CAPI) in QDS (Questionnaire Development System™). ROI investigators identified 13 domains for use across studies, informed by measures used in previous research [16–18], including questions about drug use; drug use networks; socioeconomic status; ever and past 30-day use and injection of substances to get high (heroin, street fentanyl/carfentanil, prescription opioids [e.g., oxycodone, hydrocodone, morphine, etc.], synthetic opioids [e.g., U47700/“Pink”, etc.], buprenorphine, methadone, methamphetamine, cocaine/crack, prescription anxiety drugs, gabapentin and clonidine); alcohol use and smoking; severity of dependence on opioids, methamphetamine, and cocaine/crack; access to injection equipment; drug injection and use practices; substance use treatment; criminal justice involvement; access to and utilization of health care; engagement in harm reduction programs such as utilization of SSPs, knowledge of and training on the use of naloxone, as well as access to and prior use of an overdose reversal kit; experience of stigma; HIV pre-exposure prophylaxis awareness; and diagnosis and treatment of HIV, HCV, syphilis and serious bacterial infections.

### Qualitative data

The Qualitative Working Group comprised of representatives from each study developed a core interview guide for semi-structured interviews to better understand the context of opioid and other drug use in rural settings. Guides were adapted to individual regions and administered separately from the baseline survey. Stakeholder and key-informant interviews with health departments, healthcare providers (primary care, emergency department, substance use disorder (SUD) treatment, pharmacy, etc.), law enforcement, community leaders, and other related organizations focused on: (1) local drivers of opioid use; (2) recent changes in PWUD population; (3) local barriers to addressing opioid use; and (4) suggestions for needed local changes. In addition, semi-structured interviews with PWUD covered topics such as: perceived changes in local socioeconomic conditions, substances used, and modes of administration; personal experiences with fentanyl and overdose; interactions with local law enforcement; knowledge of local laws related to naloxone rescue kits and drug paraphernalia; and use of and experiences with harm reduction, SUD treatment and SSPs.

### Laboratory data

Blood specimens were collected for rapid HIV, HCV and syphilis testing at most sites (one study used standard not rapid testing to be able to also include testing for hepatitis B virus). Specimens that were positive for HCV antibodies were forwarded to the GHOST (Global Hepatitis Outbreak Surveillance Technology) Sequencing Center at the Ragon Institute of Massachusetts General Hospital, Massachusetts Institute of Technology, and Harvard, a ROI-funded laboratory for next generation HCV sequencing, for RNA testing and sequence analysis. Linkage analyses for genetically associated transmissions and cluster detection were conducted by site among specimens with detectable RNA levels.

### Analyses

Participant characteristics are presented as counts and percentages or medians and interquartile ranges (IQR) and do not account for use of RDS recruitment weights or clustering of individuals that result from the network-based recruitment strategy. Future analyses will include multi-site qualitative analyses, evaluations of intervention impacts, and analyses that may utilize RDS-II [19] or other RDS recruitment weights as needed to reduce potential bias introduced by peer recruitment.

## Results

For each of the 8 ROI studies, the region, target population, sample size and inclusion criteria are described in Table 1. Differences in eligibility criteria were based on knowledge of the local epidemiology of opioid use and drug injection.

The ROI cohort of PWUD included 3048 individuals (range 173–991 across studies) with a median baseline age of 34 years (IQR: 28–43) (Table 2) and similar age distribution across studies. Most participants were white (85%); among the 15% who were non-white, Native American was the most common racial/ethnic group reported (7%). The cohort included 57% men, 43% women, and 1% transgender participants. Half (52%) were single, 23% had not finished high school, and 74% reported having health insurance. The percentage who reported having recently experienced homelessness was substantial: 53% in the past 6 months, although this varied across studies (36–68%).

**Table 2:** Demographic characteristics for participants in the Rural Opioid Initiative by study site, 2018–2020

	Total	Sites							
		IL	KY	NC	NE	OH	OR	WI	WV
N	3048	173	338	350	589	258	174	991	175
Age, median (IQR)	34 (28–43)	39 (31–47)	35 (29–41)	32 (27–42)	34 (28–42)	38 (32–47)	36 (29–45)	33 (27–40)	38 (32–44)
Race/ethnicity									
White	2576 (85%)	148 (86%)	331 (98%)	242 (69%)	533 (90%)	231 (90%)	145 (83%)	792 (80%)	154 (88%)
Black or African American	96 (3%)	18 (10%)	2 (< 1%)	5 (1%)	7 (1%)	13 (5%)	3 (2%)	35 (4%)	13 (7%)
Native American	225 (7%)	3 (2%)	1 (< 1%)	85 (24%)	9 (2%)	5 (2%)	9 (5%)	111 (11%)	2 (1%)
Other	148 (5%)	4 (2%)	4 (1%)	17 (5%)	40 (7%)	9 (3%)	17 (10%)	51 (5%)	6 (3%)
Unknown	3 (< 1%)	0	0	1 (< 1%)	0	0	0	2 (< 1%)	0
Hispanic^a^	116 (4%)	2 (1%)	1 (< 1%)	19 (5%)	28 (5%)	5 (2%)	16 (9%)	43 (4%)	2 (1%)
Gender									
Male	1737 (57%)	100 (58%)	193 (57%)	182 (52%)	343 (58%)	127 (49%)	99 (57%)	584 (59%)	109 (62%)
Female	1293 (42%)	73 (42%)	144 (43%)	168 (48%)	243 (41%)	130 (50%)	75 (43%)	394 (40%)	66 (38%)
Transgender/other	16 (1%)	0	1 (< 1%)	0	3 (1%)	1 (< 1%)	0	11 (1%)	0
Unknown/refused	2 (< 1%)	0	0	0	0	0	0	2 (< 1%)	0
Sexual orientation	n = 2057								
Heterosexual/straight	1771 (86%)	142 (82%)	315 (93%)	299 (85%)	485 (82%)	225 (87%)	151 (87%)	–^b^	154 (88%)
Gay/lesbian	40 (2%)	8 (5%)	7 (2%)	8 (2%)	8 (1%)	1 (< 1%)	4 (2%)	–^b^	4 (2%)
Bi-sexual/other	229 (11%)	21 (12%)	16 (5%)	39 (11%)	91 (15%)	30 (12%)	16 (9%)	–^b^	16 (9%)
Unknown/refused/missing	17 (1%)	2 (1%)	0	4 (1%)	5 (1%)	2 (1%)	3 (2%)	–^b^	1 (1%)
Marital status									
Single/not married	1570 (52%)	78 (45%)	143 (42%)	175 (50%)	326 (55%)	108 (42%)	80 (46%)	593 (60%)	67 (38%)
Separated/divorced/widowed	955 (31%)	77 (45%)	114 (34%)	114 (33%)	166 (28%)	100 (39%)	64 (37%)	247 (25%)	73 (42%)
Married	354 (12%)	15 (9%)	80 (24%)	45 (13%)	59 (10%)	43 (17%)	24 (14%)	58 (6%)	30 (17%)
Unknown/refused/missing	169 (6%)	3 (2%)	1 (< 1%)	16 (5%)	38 (6%)	7 (3%)	6 (3%)	93 (9%)	5 (3%)
Education									
Did not finish high school	688 (23%)	36 (21%)	104 (31%)	71 (20%)	153 (26%)	78 (30%)	38 (22%)	173 (17%)	35 (20%)
High school diploma or GED	1430 (47%)	64 (37%)	151 (45%)	160 (46%)	318 (54%)	114 (44%)	75 (43%)	457 (46%)	91 (52%)
Some college/Trade School	856 (28%)	68 (39%)	78 (23%)	112 (32%)	107 (18%)	65 (25%)	54 (31%)	325 (33%)	47 (27%)
College graduate or above	71 (2%)	5 (3%)	4 (1%)	7 (2%)	11 (2%)	1 (< 1%)	7 (4%)	34 (3%)	2 (1%)
Unknown/refused/missing	3 (< 1%)	0	1 (< 1%)	0	0	0	0	2 (< 1%)	0
Current Health insurance coverage	2242 (74%)	131 (76%)	277 (82%)	131 (37%)	491 (83%)	208 (81%)	146 (84%)	698 (70%)	160 (91%)
Experienced homelessness^c^	1612 (53%)	85 (49%)	123 (36%)	151 (43%)	332 (56%)	131 (51%)	119 (68%)	596 (60%)	75 (43%)

*IL* Illinois, *KY* Kentucky, *NC* North Carolina, *NE* New England (MA, NH, VT), *OH* Ohio, *OR* Oregon, *WI* Wisconsin, *WV* West Virginia

^a^Race/ethnicity are mutually exclusive categories. Hispanic includes everyone who is Hispanic. White and Black race include those who are White or Black and not Hispanic

^b^Not collected

^c^Reference period: past 6 months

Table 3 summarizes baseline substance use patterns including preferred substances for getting high and substances used in the prior 30 days, including injected drugs. Most participants identified an opioid as their preferred substance (54%). Heroin was the most common preferred opioid (38%), followed by prescription opioids (10%); only 2% reported that fentanyl or carfentanil were preferred substances. 35% reported their preferred substance was methamphetamine, with substantial variation across studies (ranging from 4% in NE to 52% in OR and WI). In the prior 30 days, 86% of participants had used opioids, 74% had used methamphetamine, 44% had used cocaine/crack, and 47% had used benzodiazepines. Polysubstance use was common: 84% reported using multiple classes of drugs in the prior 30 days, with a median of 3 drug classes. The percentage of participants reporting IDU in the past 30 days ranged from 73% to > 99% across studies. Of those who reported IDU in the past 30 days (n = 2587), 67% injected daily; the most frequently reported substances injected were methamphetamine (73%) and heroin (66%). Of note, 40% who reported injecting drugs in the past 30 days reported current injection of both opioids and cocaine (“speedball”) and/or opioids and methamphetamine (“goofball”) and 33% reported injection of fentanyl or carfentanil. In addition, in the prior 30 days, 49% of all participants reported binge alcohol use and 91% reported smoking tobacco (Table 3).

**Table 3:** Substance use patterns among participants in the Rural Opioid Initiative by study site

	Total	Sites							
		IL	KY	NC	NE	OH	OR	WI	WV
N	3048	173	338	350	589	258	174	991	175
Preferred drug for getting high									
Opioids^a^	1655 (54%)	81 (47%)	206 (61%)	171 (49%)	452 (77%)	183 (71%)	78 (45%)	378 (38%)	106 (61%)
Heroin^*^*^	1146 (38%)	34 (20%)	103 (30%)	106 (30%)	351 (60%)	124 (48%)	69 (40%)	307 (31%)	52 (30%)
Street fentanyl/carfentanil^*^*^	67 (2%)	2 (1%)	1 (< *1%)*	11 (3%)	23 (4%)	21 (8%)	0	4 (< 1%)	5 (3%)
Prescription opioids^*^*^	293 (10%)	31 (18%)	63 (19%)	44 (13%)	44 (7%)	29 (11%)	8 (5%)	36 (4%)	38 (22%)
Buprenorphine^*^*^	85 (3%)	5 (3%)	36 (11%)	4 (1%)	25 (4%)	8 (3%)	1 (1%)	0	6 (3%)
Methadone^*^*^	45 (1%)	9 (5%)	3 (1%)	6 (2%)	7 (1%)	1 (< 1%)	0	15 (2%)	4 (2%)
Methamphetamine	1070 (35%)	74 (43%)	108 (32%)	158 (45%)	23 (4%)	61 (24%)	91 (52%)	515 (52%)	40 (23%)
Cocaine/crack	188 (6%)	12 (7%)	7 (2%)	14 (4%)	95 (16%)	8 (3%)	1 (1%)	26 (3%)	25 (14%)
Benzodiazepines	39 (1%)	4 (2%)	6 (2%)	5 (1%)	4 (1%)	1 (< 1%)	1 (1%)	18 (2%)	0
Other	81 (3%)	2 (1%)	11 (3%)	2 (1%)	15 (3%)	5 (2%)	3 (2%)	39 (4%)	4 (2%)
Unknown/refused/missing	15 (< 1%)	0	0	0	0	0	0	15 (2%)	0
Drug use^b^									
Opioids^a^	2608 (86%)	144 (83%)	299 (88%)	298 (85%)	587 (99%)	241 (93%)	133 (76%)	759 (77%)	147 (84%)
Heroin^*^*^	2102 (69%)	82 (47%)	230 (68%)	230 (66%)	531 (90%)	203 (79%)	105 (60%)	605 (61%)	116 (66%)
Street fentanyl/carfentanil^*^*^	1122 (37%)	44 (25%)	95 (28%)	160 (46%)	370 (63%)	156 (60%)	19 (11%)	191 (19%)	87 (50%)
Opiate painkillers^*^*^	1744 (57%)	118 (68%)	211 (62%)	224 (64%)	339 (58%)	132 (51%)	67 (39%)	541 (55%)	112 (64%)
Buprenorphine^*^*^	1234 (40%)	84 (49%)	197 (58%)	142 (41%)	304 (52%)	116 (45%)	18 (10%)	274 (28%)	99 (57%)
Methadone^*^*^	666 (22%)	32 (19%)	51 (15%)	60 (17%)	171 (29%)	37 (14%)	27 (16%)	253 (26%)	35 (20%)
Methamphetamine	2267 (74%)	139 (80%)	265 (78%)	325 (93%)	203 (34%)	205 (79%)	168 (97%)	872 (88%)	90 (51%)
Cocaine/crack	1328 (44%)	79 (46%)	74 (22%)	82 (23%)	451 (77%)	106 (41%)	14 (8%)	432 (44%)	90 (51%)
Benzodiazepines	1433 (47%)	106 (61%)	147 (43%)	177 (51%)	300 (51%)	124 (48%)	47 (27%)	436 (44%)	97 (55%)
Other	1077 (35%)	51 (29%)	165 (49%)	77 (22%)	270 (46%)	129 (50%)	26 (15%)	277 (28%)	82 (47%)
Multiple classes of drugs used^c^	2560 (84%)	147 (85%)	294 (87%)	299 (85%)	517 (88%)	230 (89%)	137 (79%)	805 (81%)	131 (75%)
Number of classes of drugs used, median (IQR)	3 (2–4)	3 (2–4)	3 (2–4)	3 (2–4)	3 (2–4)	3 (2–4)	2 (2–3)	3 (2–4)	3 (1–4)
Ever injected drugs^d^	2812 (92%)	143 (83%)	290 (86%)	330 (94%)	499 (85%)	226 (88%)	161 (93%)	991 (100%)	172 (98%)
Recent IDU^b,e^	2587 (85%)	127 (73%)	245 (72%)	299 (85%)	431 (73%)	206 (80%)	153 (88%)	989 (> 99%)	137 (78%)
Frequency of IDU^e^									
Daily	1726 (67%)	73 (57%)	180 (73%)	222 (74%)	264 (61%)	170 (83%)	101 (66%)	629 (64%)	87 (64%)
Weekly but less than daily	483 (19%)	37 (29%)	39 (16%)	37 (12%)	84 (19%)	15 (7%)	30 (20%)	219 (22%)	22 (16%)
Less than weekly	349 (13%)	17 (13%)	26 (11%)	40 (13%)	82 (19%)	21 (10%)	21 (14%)	114 (12%)	28 (20%)
Unknown/refused/missing	29 (1%)	0	0	0	1 (< 1%)	0	1 (1%)	27 (3%)	0
IDU by drug^b,e^									
Opioids^a^	1963 (76%)	79 (62%)	207 (84%)	212 (71%)	415 (96%)	183 (89%)	92 (60%)	645 (65%)	130 (95%)
Heroin ^*^*^	1709 (66%)	61 (48%)	172 (70%)	178 (60%)	395 (92%)	169 (82%)	86 (56%)	540 (55%)	108 (79%)
Street fentanyl/carfentanil^*^*^	854 (33%)	31 (24%)	70 (29%)	120 (40%)	267 (62%)	132 (64%)	14 (9%)	148 (15%)	72 (53%)
Opiate painkillers^*^*^	845 (33%)	28 (22%)	98 (40%)	129 (43%)	116 (27%)	50 (24%)	21 (14%)	332 (34%)	71 (52%)
Buprenorphine^*^*^	642 (25%)	34 (27%)	122 (50%)	73 (24%)	115 (27%)	56 (27%)	9 (6%)	166 (17%)	67 (49%)
Methadone^*^*^	310 (12%)	13 (10%)	21 (9%)	24 (8%)	42 (10%)	16 (8%)	12 (8%)	156 (16%)	26 (19%)
Methamphetamine	1892 (73%)	110 (87%)	197 (80%)	268 (90%)	115 (27%)	169 (82%)	139 (91%)	815 (82%)	79 (58%)
Cocaine/crack	669 (26%)	32 (25%)	34 (14%)	35 (12%)	227 (53%)	47 (23%)	3 (2%)	231 (23%)	60 (44%)
Benzodiazepines	363 (14%)	18 (14%)	29 (12%)	38 (13%)	64 (15%)	24 (12%)	3 (2%)	153 (16%)	34 (25%)
Simultaneous injection of opioid & stimulant (i.e., speedball)^f^	1027 (40%)	41 (32%)	33 (13%)	160 (54%)	148 (34%)	114 (55%)	50 (33%)	408 (41%)	73 (53%)
Binge alcohol use^b^	1495 (49%)	79 (46%)	113 (33%)	154 (44%)	313 (53%)	102 (40%)	64 (37%)	584 (59%)	86 (49%)
Tobacco cigarettes^b^	2762 (91%)	159 (92%)	300 (89%)	300 (86%)	536 (91%)	240 (93%)	162 (93%)	909 (92%)	156 (89%)

*IDU* injection drug use, *IL* Illinois, *KY* Kentucky, *NC* North Carolina, *NE* New England (MA, VT, NH), *OH* Ohio, *OR* Oregon, *WI* Wisconsin, *WV* West Virginia

^a^Heroin, street fentanyl/carfentanil, prescription opioids not as prescribed, novel synthetics (i.e., U47700), buprenorphine, and/or methadone

^b^Reference period: past 30 days

^c^Use of ≥ 2 drug categories by any route in past 30 days (opioids, methamphetamine, cocaine/crack, prescription anxiety drugs not as prescribed, gabapentin, clonidine, and/or other)

^d^Injection drug use in past 30 days was an eligibility criterion at several sites, and a requirement for enrollment in WI

^e^Among participants who injected drugs in the past 30 days

^f^Simultaneous injection of opioid & methamphetamine or opioid & cocaine (i.e., speedball, goofball, or screwball)

^^^ Subcategory of Opioids; percentages among all participants

Substance-use related harms (unsafe injection, overdose and stigma) are shown in Table 4. High-risk injecting behaviors were frequently reported: among those who had injected drugs in the prior 30 days, 37% reported using a syringe/needle that had been used by someone else (range 29–55%) and 42% reported using cooker/cotton/spoons or rinses used by someone else (range 30–58%). Across studies, 49% had experienced an overdose, with a median of three lifetime (IQR: 2–5) overdoses. Only half had ever gotten an overdose kit containing naloxone or a prescription for naloxone (53%) and this also varied by region (20–72%). Most participants reported experiencing stigma, with > 75% of participants reporting feelings of shame related to drug use (range 60–85%).

**Table 4:** Substance use-related harms, engagement in harm reduction, and stigma among participants in the Rural Opioid Initiative by study site

	Total	Sites							
		IL	KY	NC	NE	OH	OR	WI	WV
N	3048	173	338	350	589	258	174	991	175
Injection practices^a,b^	n = 2587	n = 127	n = 245	n = 299	n = 431	n = 206	n = 153	n = 989	n = 137
Most common source of new syringes^c,d^									
SSP	942 (36%)	12 (10%)	93 (38%)	86 (29%)	99 (23%)	95 (46%)	38 (25%)	504 (51%)	15 (11%)
Pharmacy	454 (18%)	59 (46%)	8 (3%)	116 (39%)	113 (26%)	14 (7%)	73 (48%)	56 (6%)	15 (11%)
Friend or acquaintance	397 (15%)	27 (21%)	62 (25%)	36 (12%)	76 (18%)	28 (14%)	19 (12%)	110 (11%)	39 (28%)
Distance to nearest SSP^e^									
< 30-min drive	1928 (75%)	56 (44%)	218 (89%)	173 (58%)	274 (64%)	168 (82%)	130 (85%)	848 (86%)	61 (45%)
≥ 30-min drive	377 (15%)	9 (7%)	26 (11%)	83 (28%)	77 (18%)	27 (13%)	22 (14%)	103 (10%)	30 (22%)
Do not know where nearest SSP is located	273 (11%)	62 (49%)	1 (< 1%)	43 (14%)	79 (18%)	11 (5%)	1 (1%)	30 (3%)	46 (34%)
Used a syringe/needle used by somebody else^c^	958 (37%)	39 (31%)	76 (31%)	125 (42%)	201 (47%)	113 (55%)	48 (31%)	289 (29%)	67 (49%)
Used a cotton, cooker, spoon, or water that was used by somebody else^c^	1097 (42%)	60 (47%)	73 (30%)	159 (53%)	236 (55%)	119 (58%)	62 (41%)	318 (32%)	70 (51%)
Overdose									
Ever personally overdosed	1489 (49%)	88 (51%)	174 (51%)	178 (51%)	299 (51%)	152 (59%)	69 (40%)	439 (44%)	90 (51%)
Lifetime number of overdoses, median (IQR)^f^	3 (2–5)	2 (1–5)	2 (1–4)	3 (2–5)	2 (2–5)	3 (2–5)	3 (1–4)	3 (2–5)	3 (1–4)
Ever gotten an overdose reversal kit or prescription for naloxone or Narcan	1629 (53%)	52 (30%)	69 (20%)	221 (63%)	395 (67%)	185 (72%)	78 (45%)	551 (56%)	78 (45%)
Substance Use Treatment^g^	n = 2945	n = 166	n = 338	n = 340	n = 589	n = 256	n = 164	n = 917	n = 175
Ever received medication for OUD	1420 (48%)	59 (36%)	150 (44%)	107 (31%)	399 (68%)	162 (63%)	63 (38%)	367 (40%)	113 (65%)
Received medication for OUD in past 30 days	565 (19%)	12 (7%)	39 (12%)	26 (8%)	199 (34%)	42 (16%)	29 (18%)	150 (16%)	68 (39%)
Stigma^h^									
Feel ashamed of using drugs	2316 (76%)	127 (73%)	270 (80%)	243 (69%)	499 (85%)	215 (83%)	104 (60%)	712 (72%)	146 (83%)
Feel people avoid you because your use drugs	2094 (69%)	112 (65%)	197 (58%)	232 (66%)	450 (76%)	191 (74%)	116 (67%)	657 (66%)	139 (79%)
Fear you will lose your friends because you use drugs	1733 (57%)	93 (54%)	141 (42%)	169 (48%)	405 (69%)	161 (62%)	75 (43%)	567 (57%)	122 (70%)
Fear your family will reject you because you use drugs	2131 (70%)	118 (68%)	205 (61%)	241 (69%)	452 (77%)	188 (73%)	108 (62%)	684 (69%)	135 (77%)
Think people are uncomfortable being around you because you use drugs	1948 (64%)	105 (61%)	19 (59%)	219 (63%)	418 (71%)	170 (66%)	100 (57%)	608 (61%)	130 (74%)

*IL* Illinois, *KY* Kentucky, *NC* North Carolina, *NE* New England (MA, VT, NH), *OH* Ohio, *OR* Oregon, *OUD* opioid use disorder, *SSP* syringe service program, *WI* Wisconsin, *WV* West Virginia

^a^Among participants who injected drugs in the past 30 days

^b^Injection drug use in past 30 days was an eligibility criterion at several sites, and a requirement for enrollment in WI

^c^Reference period: past 30 days

^d^Does not add up to 100% as only most common sources listed

^e^Several studies (IL, NE, OH, OR, and WI) at least partially recruited out of SSPs

^f^Among participants who reported ever overdosing

^g^Among participants who reported ever using opioids to get high (heroin, street fentanyl/carfentanil, opiate painkillers, buprenorphine, and/or methadone)

^h^^“^Somewhat” or “Very much” vs. “Not at all” and “Just a little”

Utilization of SSPs, medication for treating opioid use disorder (MOUD) and naloxone are described in Table 4. Only 36% of PWID reported that they obtained most of their new syringes from SSPs, notable given that SSPs were recruitment sites for some studies. However, studies differed substantially, with participants from half of the regions reporting pharmacies as their typical source of syringes. More than one in four participants lived more than a 30-minute drive from the nearest SSP or did not know where the nearest SSP was located. History of having ever received MOUD was common: 48% among those who reported lifetime opioid use; however only 19% had received MOUD in the prior 30 days.

In qualitative interviews with PWUD across studies (N = 355), participants provided details contextualizing frequent concurrent methamphetamine and opioid use. Overdoses were common, and often characterized by the unintended use of fentanyl. Negative attitudes toward law enforcement were widespread. Avoiding contacting police or emergency medical services when witnessing an overdose occurred frequently. Negative prior experiences with medical providers presented a barrier to accessing health services. Additional in-depth analyses of this qualitative data will be presented in future analyses.

## Discussion

The ROI brought together researchers from eight regions to address key questions related to substance use in rural areas across the U.S. through assembling a large sample of PWUD, most of whom injected drugs. Among the cohort of 3,048 PWUD, current use was predominantly opioids (86%), however the use of methamphetamines was strikingly high (74%), and 84% reported polysubstance use in the prior 30 days. Among those with current injection drug use, a third reported injecting synthetic opioids (fentanyl/carfentanil) and more than a third (40%) reported simultaneous injection of opioids and stimulants; both practices carry high risk for overdose. Participants frequently experienced sharing syringes and other injection equipment, overdoses, homelessness, and stigma. Variations across regions highlight key differences in access to support services as well as different unmet needs.

### ROI and the current opioid crisis

The ROI consortium contributes to better understanding of patterns of opioid use in rural communities. Until recently, the U.S. opioid overdose epidemic has been characterized as having three waves [20]. The first wave followed increased opioid prescribing during the 1990s which led to increases in prescription opioid overdose deaths beginning around 1999. The second wave, beginning around 2010, reflected rapid increases in overdose deaths involving heroin. The third wave, starting around 2013, was characterized by increases in overdose deaths involving synthetic opioids (e.g., fentanyl and fentanyl analogues), while prescription opioid deaths increased only slightly, and heroin deaths stabilized. The population impacted was younger, less often male, and more likely to be white and rural than past opioid epidemics [21], although the third wave also included increases in opioid-related overdoses among Black and Hispanic PWUD in urban areas [22]. Increasing trends in HCV infections and localized HIV outbreaks among PWID [1, 23, 24] have accompanied the recent waves of opioid use in the U.S.

Findings from the ROI corroborate other recent data that suggest we have entered a fourth wave, which can be characterized as a mixed stimulant/opioid crisis [25]. Recent data from the National Survey on Drug Use and Health [26] indicated that methamphetamine use increased from 2016 to 2019 among those ≥ 26 years old. Opioid use, opioid use disorder, and initiation remained stable. Heroin use also appeared stable, while heroin initiation declined in 2019. Rates of emergency department visits for suspected nonfatal overdoses involving both opioids and amphetamines increased from 2018 to 2019 [27] and concurrent methamphetamine use among opioid users seeking substance use treatment has shown similar increases [28]. Findings from the ROI cohort illustrate this fourth wave in the variety of substances used. While opioid use still predominated, a substantial proportion also used methamphetamine, with wide variation by region.

While most of the baseline data collection ended in early 2020, our findings, particularly ongoing data collection, should be considered in the context of the COVID-19 pandemic, as social isolation, changes in drug availability and use, limited access to or outright loss of mental health and counseling services, or other factors may disproportionately impact rural communities [29]. Provisional data from the National Center for Health Statistics [30] estimates > 93,000 drug overdose deaths in the U.S. in 2020, an increase of nearly 30% compared to 2019 and the highest number of overdose deaths ever recorded for a single year; this unprecedented increase was driven by increases in overdose deaths from synthetic opioids (primarily fentanyl), methamphetamine, cocaine, and natural and semi-synthetic opioids. Some of the largest percent changes in overdose deaths from 2019 to 2020 occurred in ROI regions, including Vermont (+ 58%), Kentucky (+ 54%), and West Virginia (+ 49%) [30].

### Rural health

The findings here reflect the demographic characteristics of the local communities and the overall rural opioid epidemic, with PWUD more frequently being white and less frequently male than in previously described urban opioid epidemics [21]. Many of the communities in this cohort had experienced methamphetamine crises in the past and it was not surprising that methamphetamine use was common, with New England a major exception. Injection of opioids was not limited to heroin but included street fentanyl/carfentanil and other opioids such as buprenorphine. Access to SSPs varied widely and reflected local policies and resources. Further analysis of our qualitative data will help us understand how access occurs, particularly given the absence of public transportation and low population density in these areas [13] as well as the known disparities related to driving times for opioid treatment programs in rural vs. urban regions [31]. Rates of MOUD varied but were low, highlighting the relative lack of opioid treatment providers in rural areas. Insurance coverage was generally greater than expected; other research suggests that health care in rural areas has a greater dependency on Medicare and Medicaid reimbursement [13], which suggests that public insurance will be critical for addressing rural opioid use. The low density of credentialed behavioral health professionals and waivered buprenorphine providers in rural areas including ROI sites [32, 33], combined with often limited broadband and cell phone coverage [34], create contexts where alternatives to traditional in-person specialty care are needed but difficult to implement. Innovations in telehealth care driven by COVID-19 pandemic may provide a model for these underresourced areas.

### Strengths and limitations

ROI strengths include the large number of PWUD, primarily PWID, comprehensive and harmonized quantitative data to characterize the rural opioid epidemic, rich qualitative data from PWUD to provide context, and stakeholder interviews to better understand available local resources and needs. To our knowledge, this study offers the largest and most geographically diverse sample of rural people who use opioids to date; importantly, it reflects the fourth stage of the opioid overdose epidemic, characterized by both opioid and stimulant use [25]. Nonetheless, better characterization of the patterns of overlapping opioid and stimulant polysubstance use and resulting overdoses are still needed. The ROI brings together a multidisciplinary group of researchers, substance use treatment providers, public health experts and others to provide expertise to improve benefits across all areas studied. The standardized data collection process and measures across studies allows data harmonization that enhances an overall understanding of the opioid crisis and related comorbidities in rural areas. Limitations include the potential that these eight regions may not necessarily represent the opioid- and IDU-related experiences of all U.S. rural regions, and that respondent-driven sampling may not have recruited a representative sample. However, core areas of the current crisis– including central Appalachia, the rural Midwest, and New England– are represented along with historically methamphetamine-impacted areas of the Pacific Northwest.

### Next steps

A key goal of phase one data collection was to identify potential areas of intervention. Phase two data collection is underway and involves seven different interventions tailored to specific needs. The interventions range from expansion of harm reduction services in underserved areas to randomized, multicomponent interventions. The most common components of the interventions are (1) telehealth, predominantly for HCV treatment; (2) peer navigation to increase testing and linkage to care for HIV, HCV and SUD; and (3) community capacity-building and engagement to reduce stigma and enhance provider capacity. It is worth noting that variation in interventions was intended to ensure that they were contextually relevant and driven by data-informed needs of each region.

## Conclusions

The ROI represents an unprecedented collaboration among federal agencies, researchers, and public health stakeholders spanning multiple jurisdictions throughout the U.S. dedicated to understanding and addressing the syndemic of infectious diseases and drug use in rural settings. By combining data across studies, the ROI provides much greater statistical power to address research questions and better understand drivers and potential interventions in rural areas where resources are more limited and stigma remains a key obstacle. The data also provide an opportunity to examine community assets critical to rural PWUD’s resilience in the face of obstacles. With data from > 3,000 rural PWUD from eight regions, the ROI provides a number of clinical insights including demonstrating the current overlap between opioid and methamphetamine use, frequent homelessness, tremendous stigma and other unmet needs. Knowledge gained from the ROI has informed the development and current evaluation of public health interventions to reduce the morbidity and mortality associated with opioid and other drug use that may be implemented in underserved rural settings.

## Data Availability

We welcome collaboration and encourage mentorship and the use of the ROI data stripped of all protected health information (PHI) to enable early investigators to address meaningful questions with support to help ensure their success. Additional information can be obtained at the ROI website: ruralopioidinitiative.org or by contacting the ROI DCC at ruralopioidinitiative@uw.edu. Follow the Rural Opioid Initiative on Twitter @ruralopioids.

## Abbreviations

ACASI: Audio computer-assisted self-interview
ARC: Appalachian Regional Commission
CAPI: Computer-assisted personal interview
CASI: Computer-assisted self-interview
CDC: Centers for Disease Control and Prevention
DCC: Data coordinating center
HCV: Hepatitis C virus
IDU: Injection drug use
IL: Illinois
KY: Kentucky
MOUD: Medication for opioid use disorder
NC: North Carolina
NE: New England
NIDA: National Institute on Drug Abuse
OH: Ohio
OR: Oregon
PWID: People who inject drugs
PWUD: People who use drugs
QDS: Questionnaire Development System
RDS: Respondent-driven sampling
ROI: Rural Opioid Initiative
SAMHSA: Substance Abuse and Mental Health Administration
SSP: Syringe service program
SUD: Substance use disorder
US: United States
WI: Wisconsin
WV: West Virginia
MA: Massachusetts
NH: New Hampshire
VT: Vermont
CT: Connecticut
